# Immature Stages of the Neotropical Cracker Butterfly, *Hamadryas epinome*

**DOI:** 10.1673/031.012.7401

**Published:** 2012-07-04

**Authors:** Luis Anderson Ribeiro Leite, Fernando Maia Silva Dias, Eduardo Carneiro, Mirna Martins Casagrande, Olaf Hermann Hendrik Mielke

**Affiliations:** ^a-e^ Departamento de Zoologia; Setor de Ciências Biológicas; Universidade Federal do Paraná; C. P.: 19020; 81531– 980, Curitiba, Parana, Brazil

**Keywords:** bionomy, chaetotaxy, life cycle, Papilionoidea

## Abstract

The external morphology of the immature stages of *Hamadryas epinome* (C. Felder & R. Felder, 1867) (Lepidoptera : Nymphalidae : Biblidinae) is described, including drawings, photos and scanning electron micrographs.

## Resumo

A morfología externa dos estágios imaturos de *Hamadryas epinome* (C. Felder & R. Felder, 1867) (Lepidoptera: Nymphalidae: Biblidinae) é descrita, incluindo desenhos, fotos e microscopia eletrônica de varredura.

## Introduction

*Hamadryas epinome* (C. Felder & R. Felder, 1867) is a Neotropical species restricted to South America. It has been found in the area stretching from the northeastern Brazilian coast to Uruguay, as well as regions of Peru, Bolivia, Paraguay and northern Argentina (Jenkins 1983; Contreras Chialchia & Contreras Roque 2010). According to Jenkins (1983), some Central American distributional records are possibly inaccurate due to labeling errors.

During its larval instars, *H. epinome* is associated with plants belonging to the genera *Dalechampia* (Euphorbiaceae) and *Inga* (Fabaceae); however, the latter considered a doubtful association by a many (Jenkins 1983; Canals 2003; Pastrana 2004; Contreras Chialchia & Contreras Roque 2010).

Several studies include immature Neotropical Nymphalidae (Casagrande 1979; Casagrande & Mielke 1985, 2000; Paim et al. 2004; Casagrande & Mielke 2005; Freitas 2006; Silva et al. 2006; Kaminski et al. 2008; Dias et al. 2010). However, there is little published information about the subfamily Biblidinae (Freitas et al. 1997). While some immature *Hamadryas* Hübner, [1806] have been described in previous literature (e.g. Young 1974; Muyshondt & Muyshondt 1975a; 1975b; 1975c), other species remain completely unknown, or have limited information available about them *(e.g.* Müller 1886; D'Almeida 1922), such as only their adult or host plant, or their geographical distribution (Jenkins 1983; DeVries 1987; Neild 1996; Contreras Chialchia & Contreras Roque 2010). Immature *H. epinome* were briefly described, but with no available illustrations, by Müller (1886) and D'Almeida (1922).

Research on the morphology and natural history of immature stages is highlighted in literature as a fundamental tool for taxonomic and evolutionary studies of Lepidoptera, especially at higher taxonomic levels (Freitas et al. 1997; Greeney & Gerardo 2001; Hasenfuss & Kristensen 2003; Casagrande & Mielke 2005; Silva et al. 2006; Souza et al. 2006; Freitas & Brown Jr. 2008; Dias et al. 2010; Leite et al. 2010). With the objective of contributing to the systematic studies of the subfamily Biblidinae, this study describes the external morphology and behavioral aspects of the immature stages of *H. epinome.*

## Materials and Methods

Eggs were collected in the Barigui Municipal Park (25° 25′ S, 49° 18′ W, 910 m a.s.l.), Curitiba, Paraná, Brazil, together with the host plant *Dalechampia triphylla* Lam. (Euphorbiaceae). Females were observed ovipositing underneath the leaves, which allowed for accurate identification of specimens. Several *Inga* plants were examined throughout adjacent regions, but no eggs were found. Collected specimens were maintained and reared in the laboratory. Initially, eggs were kept together with the leaves on which they were laid, on absorbent paper in Petri dishes, where the humidity was maintained by daily water spraying. After eclosion, the larvae were transferred to plastic rearing cages, where they were observed whenever the *D. triphylla* leaf twigs were changed. The leaf twigs were changed daily, or when they had wilted. The specimens were examined daily in order to record behavioral observations, and to search for exuviae. Data on eclosion, instar change, pupal duration and emergence were recorded. Each new event was photographed using a digital camera, “automontage” techniques, and scanning electron microscopy. A minimum of three larvae were preserved for each larval stage and instar, and were sacrificed in boiling water, fixed in a 10% Kahle-Dietrich solution for three days and preserved in 70% alcohol. Cephalic capsules and pupal exuviae were kept dry inside plastic containers. Drawings and measurements were made using a stereoscopic microscope equipped with a camera lucida and micrometric objectives, except for the drawings and measurements of the first instar head capsule chaetotaxy, which were made by using a microscope equipped with a camera lucida. The terminology of Dell'Erba et al. (2005) was followed for the eggs, Hinton (1946), Peterson (1962), Stehr (1987), Dias (2006), and Huertas Dionisio (2006) for the larval structures, and Mosher (1916) and Dias (2006) for the pupal structures. Immature and adult specimens were deposited in the Pe. Jesus Santiago Moure Entomological Collection of the Universidade Federal do Paraná.

## Results

**Egg** (Figures 5, 6, 23–25). Elliptical, ornamented with numerous irregular vertical carinae, with a flattened bottom pole. Uniform pearly white color, gradually becoming transparent while nearing hatching. Micropylar region evident in the upper pole within a rounded and slightly concave area. Isolated placement on the adaxial or abaxial surface of the leaf. Mean diameter: 0.88 mm. Average duration: 08 days (n = 10).

**First instar larva** (Figures 7, 8, 26–35). Head rounded, black and without scoli or ornamentation. Anterior region divided by the epicranial suture. Lateral to the anterior region, the epicranium occupies the largest portion of the head, and is where the majority of the primary setae are found. Frons triangular, medially located, laterally delimited by the epicranial suture, and inferiorly delimited by the clypeus, which has the shape of a sclerotized transverse band. Labrum bilobed. Mandibles strongly sclerotized with a jagged cutting edge. Six stemmata located on the latero-inferior region, with 1–4 and 6 arranged in a semi-circle. Stemmata 5 is ventral to the others and closer to the base of the antenna, which has three antennomeres. The basal antennomere is larger and wider, the median one is smaller, and the distal one is elongated, with distal setae.

Pronotal plate divided and black, forming two sub-rectangular regions, with four pairs of setae on chalazae. Thoracic legs similar, the same color as the pronotal plate, consisting of a thigh, trochanter, femur, tibia, and an unsegmented tarsus with a terminal claw. Integument of the rest of the body greenishyellow, with white rounded spots regularly distributed dorsally and laterally. Numerous black setae distributed throughout the body, sometimes spiniform, sometimes clavate, the vast majority on chalazae, with some belonging to the thoracic segments and arranged in verrucae. Bases of all black setae forming circular blackened areas interspersed with white rounded areas. The T1 and A8 spiracles elliptical and similar in size, other spiracles smaller and rounded. The A3-A6 prolegs with crochets arranged in a uniordinal circle, and the A9 and A10 prolegs with crochets in a uniordinal penellipse. After eclosion, first instar larvae remained isolated, feeding independently, often not sharing the same leaf. Head capsule: 0.64 mm width, 0.56 mm height. Average length of the larva before moulting: 4.94 mm. Average duration: 04 days (n = 9)

**Chaetotaxy of head capsule** (Figures 28–31). Twenty-one pairs of primary setae on chalazae, excluding those belonging to the labrum. Clypeal Group (C): Spiniform setae, both with the same length, and located lateroinferiorly on the clypeus; C1 lateroventral to C2, and closer to the epicranial suture. Frontal group (F): F1 spiniform of reduced size; Fa pore at the same height as F1, and near the sagital midline. Adfrontal Group (AF): AF1 dorsal to AF2 in the adfrontal area, both spiniform, and similar in length; AFa pore closer to AF2. Anterior Group (A): A1 spiniform, and near the bottom edge of the epicranium; A2 clavate, smaller, and dorsal to A1, located between the stemmatal region and the epicranial suture; A3 clavate and dorsal to the stemmatal region, the largest among the anterior setae; Aa pore dorsolateral to stemmata 1. Stemmatal Group (S): Setae clavate, and of the same length; S1 lateral to stemmata 3; S2 dorsolateral to stemmata 1; S3 in the posterior region of the epicranium, lateral to the stemmatal semicircle; Sa pore ventrolateral to stemmata 4. Substemmatal Group (SS): Setae spiniform, and of the same length in the posterior-inferior region of the epicranium; SS1 near the mouthparts; SS2 ventral to the SSb pore and between SS1 and SS3; SS3 lateral to stemmata 5; Sb pore ventrolateral to stemmata 6. Lateral Group (L): L1 clavate, and dorsolateral to A3; La pore ventrolateral to A3. Posterodorsal group (P): Setae clavate; P1 ventral to P2, and nearest to epicranial suture; Pb pore between P1 and P2, and closer to the latter; Pa pore in the mediolateral region of the epicranium, and near A3. Microdorsal group (MD): Tiny, located in the posterior dorsal region of the head; MD1 ventral to the others, and closer to the occipital foramen; MD2 ventrolateral to MD3; MDa pore lateral to MD3. Microgenal group (MG): MG1 tiny, close to the occipital foramen; MGA pore ventrolateral to MG1.

**Chaetotaxy of thorax** (Figs 32, 33). Prothorax: Ten pairs of setae. D1 arranged in anterior-dorsal margin of pronotal plate; D2 ventral to D1 in the posterior margin; Xd1 and Xd2 ventral to D1, all pronotal plate setae clavate. Subdorsal Group (SD) ventral to pronotal plate with setae arranged in a verruca, SD1 clavate and anterior to SD2, the latter being spiniform. Lateral Group (L) in a verruca ventral to first spiracle, L2 anterior and spiniform, L1 clavate. Supraventral Group (SV) in a verruca ventral to lateral setae, SV2 spiniform and anterior to SV1, the latter clavate. Meso and Metathorax: Six pairs of clavate setae, except SD2. D1 dorsal to D2; SD1 and SD2 arranged in a verruca ventral to D2, SD1 clavate and anterior to spiniform seta SD2. L1 ventral to SD2 and SV1 ventral to L1.

**Chaetotaxy of abdomen** (Figures 32, 34, 35). A1: Seven pairs of clavate setae, except for ventral seta V1, which is spiniform; D1 anterodorsal to D2, and SD1 ventral to the dorsal group setae; L1 posterodorsal to L2; SV1 ventral to lateral setae and SV1. A2: Distribution similar to A1, except for the presence of SV2 dorsal to V1. A3-A6: Eight pairs of setae distributed as in A2, except for the Supraventral group (SV) setae, which are both spiniform, with SV2 being smaller than, and anteroventral to, SV1. A7: Distribution similar to A1. A8: Six pairs of setae with arrangement similar to A1 and A7, except for the absence of V1. A9-A10: Eighteen pairs of clavate or spiniform setae. In the A9 region, D1 anterodorsal to D2; SD1 ventral to D2; L1 posteroventral to SD1; SV1 spiniform, and anteroventral to L1. In the A10 region, D1 on the dorsal margin of the anal plate; D2 ventral to D1; SD1 located on the anteroventral margin of anal plate; SD2 spiniform, on the posteroventral margin, and closer to D2. The setae belonging to the posteroventral region of A10 are spiniform; PP dorsal to SV1on the paraproct; L1, L2, L3, SV2, and SV3 arranged in a verruca; SV4 anteroventral to SV3; V1 ventral to verruca on the median line between SV3 and SV2.

**Second instar larva** (Figures 9, 10, 36). Head capsule black. Epicranium with a pair of short dorsal scoli, which appear truncated, with setae distributed on chalazae. Setae on lateral and lateroventral regions of the epicranium, on cream-colored chalazae. Remaining body segments dark brown, with a narrow light brown lateral band on all the segments, at the level of the spiracles. Body covered with either yellowish or blackened scoli, the latter having a creamy distal region, with the color of the scoli alternating along the segments. A3-A6 and A10 prolegs with crochets arranged in a uniordinal penellipse. Head capsule: 0.93 mm width, 0.82 mm height, 0.4mm length of dorsal protuberance. Average length of the larva before moulting: 5.88 mm. Average duration: 4 days (n = 7).

**Third instar larva** (Figures 11, 12, 37). Head capsule black. Dorsal scoli longer and slender when compared to previous instar, with a blackened basal half, and composed of strong spiniform setae having a light brown distal half, and a blackened globose apex with many minute setae on chalazae. Thoracic and abdominal segments, as well as the scoli distributed on the body, with the same coloration as the head, except for some lateral scoli of the T2 to A8, which are cream colored. Prolegs with crochets similar to the previous instar. Head capsule: 1.2 mm width, 1.14 mm height, 2.58 mm length of dorsal scoli. Average length of the larva before moulting: 13.1 mm. Average duration: 4 days (n = 7).

**Fourth instar larva** (Figures 13, 14, 38). Head capsule similar to previous instar. Prothorax with an orange brown anterior region, and a black pronotal plate. Posterior region of the prothorax, along with other segments, grayish brown. An orange brown stripe laterally along T2 and T3, and on the abdominal segments at the level of the spiracles. Prolegs of A3-A6 and A10 with crochets arranged in a biordinal penellipse. Head capsule: 1.97 mm width, 1.6 mm height, 4 mm length of dorsal scoli. Average length of the larva before moulting: 22.3 mm. Average duration: 4 days (n = 7).

**Fifth instar larva** (Figures 15, 16, 39, 40). Head capsule similar to third and fourth instars. Pronotal plate with the same coloration as the head. Spiniform setae distributed throughout the body, either grouped in scoli, or individualized (Fig. 40). Setae color similar to head, except for those belonging to the A1 to A8 supraventral and ventral regions, which are yellowish. Thorax and abdomen tegument, with the same color as the head and setae on the dorsal, subdorsal and lateral regions. Two pairs of yellow longitudinal stripes dorsally from the T2 to A8, with those closer to the median longitudinal line being more conspicuous than the others. The orange brown band placed laterally, as in the previous instar. Prolegs with crochets similar to previous instar. Head capsule: 3 mm width, 2.97 mm height, 6.47 mm length of dorsal scoli. Average length of the larva before moulting: 31.5 mm. Average duration: 8 days, including one day in prepupa, (n = 7).

**Pupa** (Figures 17–22, 41–43). Adecticous, obtect and suspended by the cremaster, attached to silk woven on twigs of host plant. Pupa with elongated aspect, gradually diminishing in direction of the cremaster, dorsally projected on T2 and A2, forming crests. Thin tegument, initially of light green color ventrally, olive green dorsally, and, after a minimum twenty four period, reaches a light brown tone ventrally, and a greenish brown tone dorsally. Light brown dorsolateral band from the vertex to the lateral margin of A9. Longitudinal median light brown band, which is narrow in T1 and T2, and widened from T3 to A9.

Head vertex with a pair of foliaceous dorsolateral appendices. Frons with a smooth aspect, sub quadrangular clypeus. Conspicuous mandibles inferior lateral to the clypeus. Small pentagonal labrum, inferior to the clypeus and between the mandible. Galeae arising inferiorly to the mandibles, and inferior lateral to the labrum, ending next to the distal extremity of the antennae. Eyes lateral to the mandibular region, and with an oval format. Antennae with striated aspect, lateral to the eyes, and at the base of the foliaceous appendices, ending close to the galeae along the margins of the anterior wings.

Subrectangular pronotum with a median suture, and smaller in relation to the other thoracic segments. Mesonotum larger among the three segments. Metanotum with an anteromedian convex margin. Prothoracic legs close to the anterior third of the antennae, and terminating on its posterior third.

Abdomen with ten segments, elliptical spiracles laterally distributed from A2 to A8, with the A8 pair smaller than the other. A thin, dark brown line ventrally from A4 to A8. Cremaster darkened, on apex of A10, with simple distal hooks of dark brown color.

Average length of the pupa in both sexes, from the vertex to the cremaster: 17.64 mm. Average length of the foliaceous appendices: 7.64 mm. Average duration: 8 days, (n=6).

## Discussion

Despite the previous publication of many descriptions of immature Nymphalidae, knowledge of systematically relevant morphological information remains scarce. This scarcity of knowledge is partly because the first descriptive works are old, and their illustrations and descriptions are scarce, superficial, and incomplete. Furthermore, the first descriptive works primarily concentrated on the natural history aspects of each species, many times in detriment of comparative morphology aspects. For this reason, detailed comparative studies, including chaetotaxy, morphology of scoli, and head capsules, or ultra-structure, are still scarce.

With respect to host-plants, *Hamadryas* species appear to feed on *Dalechampia,* considering that the majority of their species feed exclusively on this Euphorbiaceae genus (Muyshondt & Muyshondt 1975a; 1975b; 1975c; Jenkins 1983). In contrast, no observation of *Hamadryas* feeding on *Inga* has been made for the region, in spite of the examination of many plants. In addition, adults of other species of the genus (e.g. *Hamadryas iphthime* (H. W. Bates, 1864), *Hamadryas fornax fornax* (Hübner, [1823]), *Hamadryas* amphinome amphinome (Linnaeus, 1767), *Hamadryas februa februa* (Hübner, [1823])) were reared in breeders containing both plants, but there was no oviposition on *Inga* spp. Therefore, the discussion in Jenkins (1983), where the feeding records on *Inga* spp. were stated to be identification errors, must be reliable.

The irregular ornamentation of the chorion on the eggs appears to be common among *Hamadryas* species. In contrast, the eggs of *Panacea* Godman & Salvin, 1883 and *Batesia* C. Felder & R. Felder, 1862 have defined crests, such as are found in other Biblidinae, which normally present well defined carenae (DeVries et al. 2000; Daniels et al. 2008). Unfortunately the eggs of *Ectima* Doubleday, [1848], a genus commonly referred to as a sister group of *Hamadryas* (Freitas & Brown Jr. 2004; Wahlberg et al. 2009) have not been illustrated up to this date. More studies must be done to analyze the patterns of the carenae on the different groups of Biblidinae.

During the fifth larval instar, the predominance of long scoli distributed along and covering the whole body contrasts with the small scoli found in some species of *Callicore* Hübner, [1819], *Perisama* Doubleday, 1849, *Haematera* Doubleday, 1849, *Cybdelis* Boisduval, 1836, and *Eunica* Hübner, [1819] (Barbosa et al. 2010). Developed scoli would be characteristic of Ageroniini, Catonephelini, and Eubagini species (Wahlberg et al. 2009). The absence of dorsal scoli between the segments A2 and A8 was described as a differentiating characteristic of *H epinome* (Müller 1886), and was later repeated in other comparative studies (Muyshondt & Muyshondt 1975a, 1975b, 1975c; Jenkins 1983). This characteristic is also cited for *H*. *guatemalena, H fornax,* and *H. amphinome* (Muyshondt & Muyshondt 1975a, 1975b, 1975c). Although the presence of this characteristic has not been confirmed for these species, in *H. epinome,* the dorsal scoli are present in all the segments, and smaller than the subdorsal ones, More refined studies on the morphology of the other species should be done in order to verify if these differences occur due to the form of the scoli, or if they are simply observation errors.

The foliaceous aspect of the extensions in the anterior region of the pupae are not only informative between species of *Hamadryas,* but also for Biblidini in general. In Biblidini, different forms and lengths of extensions are observed. The extensions can be absent or reduced (Muyshondt 1973; Torres Bauzá 2000; Barbosa et al. 2010), or long and leafy, as in the species of *Hamadryas* (Muyshondt & Muyshondt 1975a, 1975b, 1975c) and *Ectima* (Janzen 2010). In Ageroniina (Lamas 2004), the pupae of *Batesia* and *Panacea* species are radically different from *Hamadryas* and *Ectima,* possessing no extensions on the head, and having a creamy yellow coloration with dark spots distributed throughout the body (DeVries et al. 2000; Daniels et al. 2008). Beyond the foliaceous aspect of the extensions, *Ectima* species also share the brown coloration, and the elongated and slender characteristics with *Hamadryas* species. Among the *Hamadryas* species, the projections of the head can be partially fused, or have their apex gradually curved (Muyshondt & Muyshondt, 1975a, 1975b, 1975c).

Although at this time there is no hypothesis on the phylogenetic relationship between the *Hamadryas* species, it is assumed that the group presents three species groups, whose differences are mostly present in adult stages only (Jenkins 1983). However, the diversity of characteristics found in the immature stages suggests that the integration of adult stage and immature stage information is fundamental to the understanding of the systematics of this genus.

**Figures 1–4.**
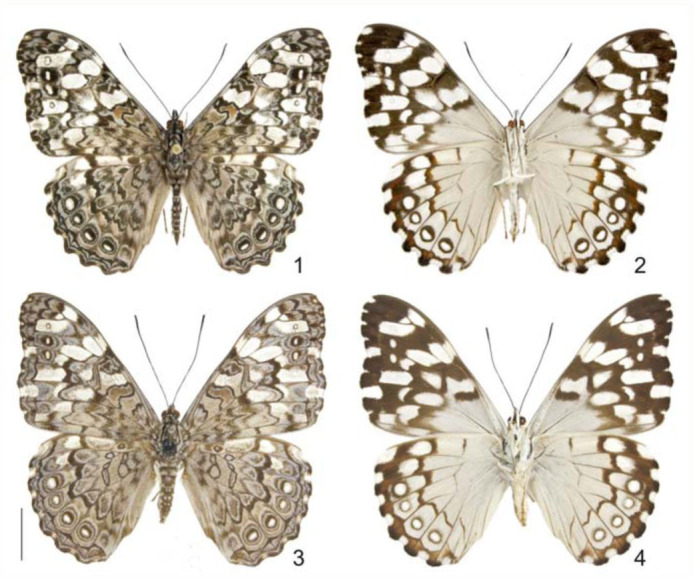
*Hamadryas epinome.* 1, 2. Male: 1, dorsal view; 2, ventral view; 3, 4. Female: 3, dorsal view; 4, ventral view. Scale bar = 1 cm. High quality figures are available online.

**Figures 5–16.**
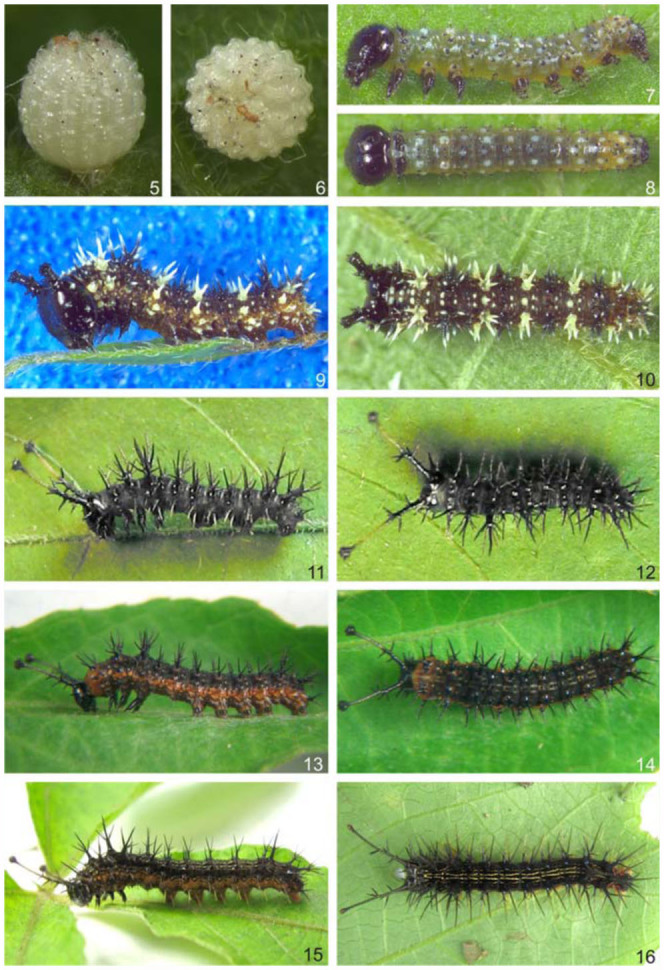
*Hamadryas epinome.* 5, 6. Egg: 5, lateral view; 6, dorsal-superior view; 7–1 6. Larvae: 7, 8. 1st instar: 7, lateral view; 8, dorsal view; 9, 10. 2^nd^ instar: 9, lateral view; 10, dorsal view; 11, 12. 3^rd^ instar: 11, lateral view; 12, dorsal view; 13, 14.4^th^ instar: 13, lateral view; 14, dorsal view; 15, 16. 5th instar: 15, lateral view; 16, dorsal view. High quality figures are available online.

**Figures 17–22.**
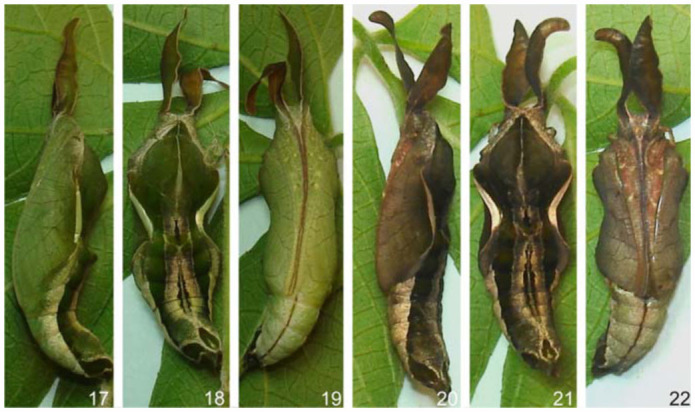
*Hamadryas epinome.* 17–19. Early pupae: 17, lateral view; 18, dorsal view; 19, ventral view; 20–22. Pupae 24 hours later: 20, lateral view; 21, dorsal view; 22, ventral view. High quality figures are available online.

**Figures 23–27.**
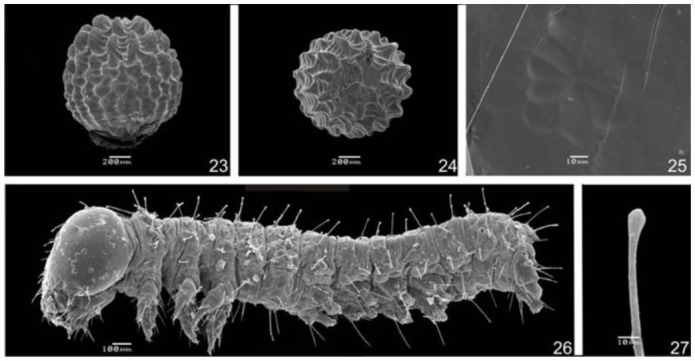
*Hamadryas epinome.* 23–25. Egg: 23, lateral view; 24, dorsal superior view; 25, micropilar region; 26, 27. 1^st^ instar larva: 26, lateral view; 27, dorsal scoli of the 1^st^ instar larva. High quality figures are available online.

**Figures 28–31.**
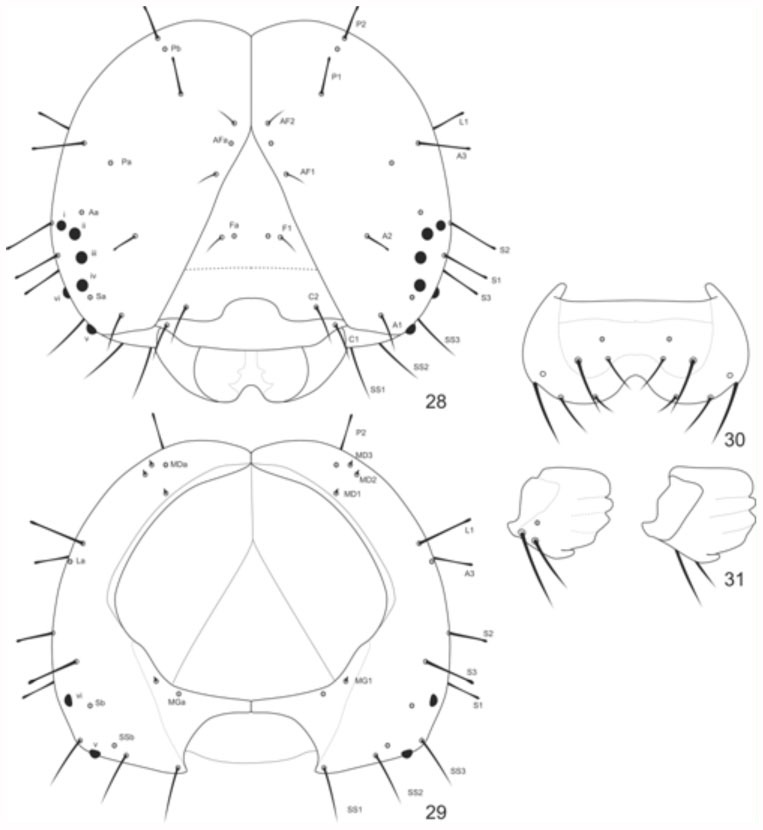
*Hamadryas epinome.* 1^st^ instar larva: Head chaetotaxy: 28, frontal view; 29, posterior view; 30, labrum; 31, mandibule. High quality figures are available online.

**Figures 32–35.**
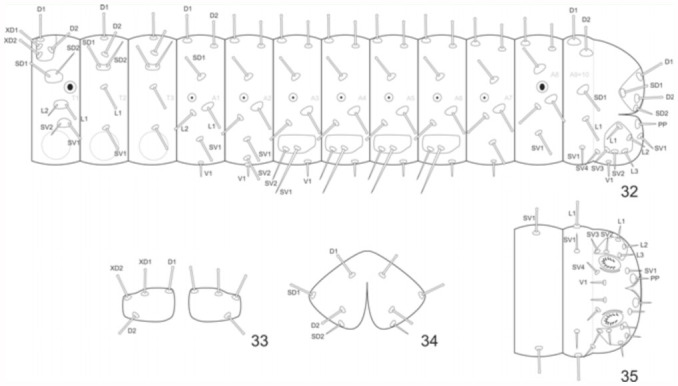
*Hamadryas epinome.* 1^st^ instar larva: 32, thorax and abdomen chaetotaxy; 33, pronotal plate chaetotaxy; 34, anal plate chaetotaxy; 35, chaetotaxy of the ventral region A8-A10. High quality figures are available online.

**Figures 36–37.**
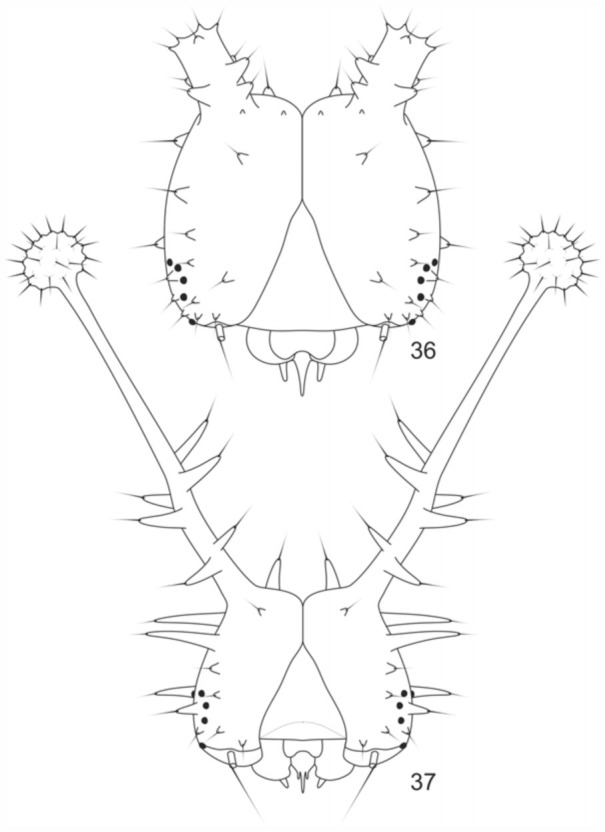
*Hamadryas epinome.* 2^nd^ and 3^rd^ instar larvae: Head capsules: 36, frontal view of the 2^nd^ instar; 37, frontal view of the 3^rd^ instar. High quality figures are available online.

**Figures 38–39.**
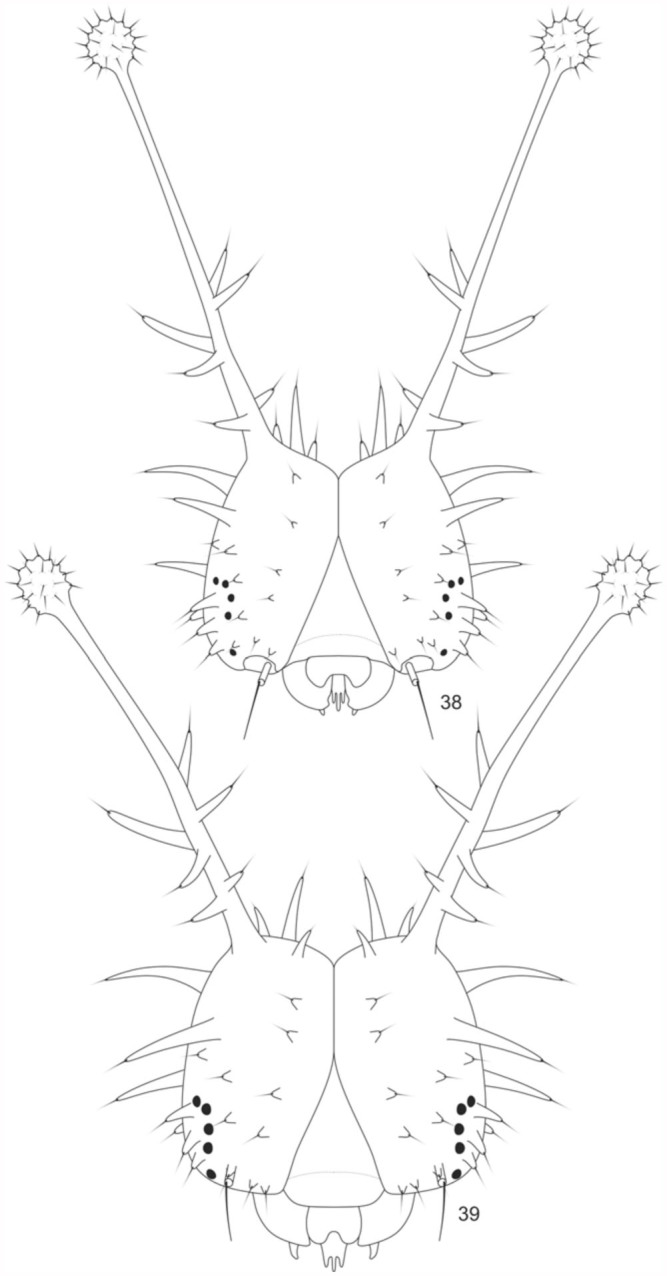
*Hamadryas epinome.* 4^th^ and 5^th^ instar larvae: Head capsules: 38, frontal view of the 4^th^ instar; 39, frontal view of the 5^th^ instar. High quality figures are available online.

**Figure 40.**
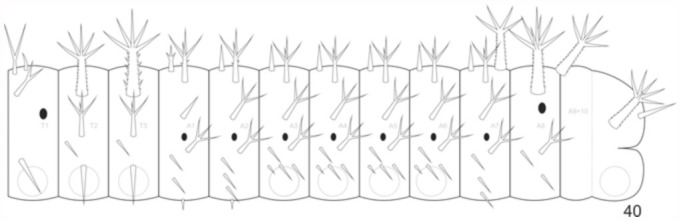
*Hamadryas epinome.* 5^th^ instar larva: thorax and abdomen chaetotaxy. High quality figures are available online.

**Figures 41–43.**
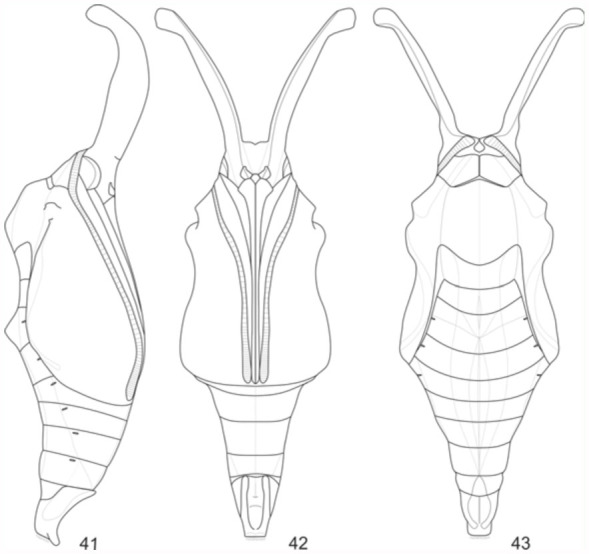
*Hamadryas epinome.* Pupae: 41, lateral view; 42, ventral view; 43, dorsal view. High quality figures are available online.
